# Delayed Recognition of Multiple Small Bowel Perforations and Mesenteric Lacerations Following Combined Blunt and Penetrating Lumbar Trauma: A Case Report

**DOI:** 10.7759/cureus.114322

**Published:** 2026-08-11

**Authors:** George O Mallouka, Kareem Hanna, Veronica E Tawadros, Muhamed S Zubedi, Joaquin Picazo-Yeste

**Affiliations:** 1 General Surgery, Sheikh Khalifa Medical City, Abu Dhabi, ARE; 2 Medicine, Royal College of Surgeons in Ireland - Bahrain, Busaiteen, BHR

**Keywords:** blunt abdominal trauma, case report, delayed perforation, mesenteric injury, penetrating trauma, small bowel injury

## Abstract

Small bowel and mesenteric injuries (SBMI) following abdominal trauma are challenging to diagnose, particularly when initial imaging is non-definitive and multiple distracting injuries are present. Bowel injury may remain occult during the initial assessment, and delayed recognition can result in significant morbidity and mortality.

A 39-year-old previously healthy male construction worker sustained a penetrating injury to the right lumbar region after a heavy object fell from approximately 2-2.5 meters, with retained steel fragments and associated blunt abdominal trauma. He was hemodynamically stable on presentation, with a negative focused assessment with sonography for trauma (FAST) examination. Initial computed tomography (CT) demonstrated a right psoas intramuscular hematoma containing small air locules with surrounding soft tissue stranding. No pneumoperitoneum, mesenteric hematoma, or definitive radiological evidence of hollow viscus injury was identified. After wound debridement, he was admitted for close observation. His abdominal symptoms initially improved, but on post-injury Day 3 he developed worsening abdominal pain. Examination at that time showed abdominal distension, rigidity, newly developed abdominal wall bruising, and palpable subcutaneous crepitus. Repeat CT demonstrated significant pneumoperitoneum and peritonitis. Diagnostic laparoscopy was performed and converted to laparotomy following identification of intestinal content. At laparotomy, there was diffuse four-quadrant peritonitis with multiple distal ileal perforations and mesenteric lacerations. The involved ileal segment was resected, followed by stapled side-to-side anastomosis, washout, and drain placement. The patient recovered without major complications and was discharged on postoperative day nine.

This case highlights the limitations of initial CT imaging in detecting bowel injury, particularly in combined trauma involving retroperitoneal and musculoskeletal injuries. The exact timing of the bowel perforations in this patient could not be established, and the injuries may have been initially occult before becoming clinically apparent. Serial clinical monitoring and a low threshold for repeat imaging are critical. Early repeat imaging or diagnostic laparoscopy should be considered in patients with persistent or concerning abdominal findings despite non-definitive initial imaging.

A high index of suspicion for SBMI should be maintained following combined blunt and penetrating trauma, even when initial imaging is non-definitive. Continued clinical reassessment and timely further investigation are important when abdominal findings persist or recur.

## Introduction

Small bowel and mesenteric injuries (SBMI) are uncommon but serious consequences of blunt abdominal trauma, with an incidence of approximately 1-5% [1,2]. Missed or delayed diagnosis may result in peritonitis, sepsis, and death [3-5]. Although penetrating and blunt mechanisms are well described independently, combined mechanisms can present additional diagnostic complexity when the trajectory and extent of injury are not immediately apparent. High-energy blunt force may be transmitted across the abdomen, producing compression of the bowel against the abdominal wall and vertebral column and resulting in bowel or mesenteric injury [6].

Computed tomography (CT) is the primary imaging modality in hemodynamically stable trauma patients, but it does not identify every bowel or mesenteric injury. Reported sensitivity ranges from 75 to 95%, with specificity of 85-100% [7-10], and up to 20-30% of SBMI may initially be missed when findings are subtle or obscured by adjacent hematomas or associated injuries [3,11].

Traumatic bowel injury may also evolve through progressive impairment of blood supply and ischemia, hematoma expansion, or bowel wall injury that is not apparent during the initial assessment [3,11]. This case report describes the delayed recognition of multiple small bowel perforations and mesenteric lacerations following combined blunt and penetrating lumbar trauma.

This case report has been reported in line with the SCARE 2025 criteria [12].

## Case presentation

A 39-year-old previously healthy male construction worker was brought to the emergency department after a heavy object fell from approximately 2-2.5 meters onto his right lumbar region. The injury involved both penetrating trauma with retained fragments and blunt abdominal impact after falling. He complained of severe generalized abdominal and right lumbar pain (10/10). There was no loss of consciousness, vomiting, or neurological deficit. Past medical history was unremarkable. He was not on regular medications, had no known allergies, and had no history of smoking or alcohol consumption. 

Upon arrival to the emergency department, a trauma code was activated. The patient was hemodynamically stable with blood pressure 165/92 mmHg, heart rate 94 beats/min, respiratory rate 14 breaths/min, temperature 36.9°C, and oxygen saturation 99% on room air. Airway was patent, breathing was symmetrical, circulation was stable, and focused assessment with sonography for trauma (FAST) examination was negative. The Glasgow Coma Scale was 14/15 with mild disorientation.

During the primary trauma survey, the abdomen was generally firm with diffuse tenderness and guarding. On subsequent examination, the abdomen was soft and non-distended with generalized tenderness but no guarding. The general surgery team's assessment later documented mild generalized tenderness without guarding. No abdominal wall bruising or wounds were visible at presentation. A deep right lumbar laceration measuring approximately 7 cm in depth was identified.

Initial laboratory investigations showed leukocytosis with neutrophilic predominance as seen under "Day 0" in Table 1. Initial venous blood gas analysis showed a pH of 7.30 (reference range: 7.35-7.45), pCO₂ of 47.5 mmHg (reference range: 35.0 - 45.0 mmHg), and lactate of 1.9 mmol/L (reference range: 0.50 - 2.20 mmol/L).

**Table 1:** Laboratory investigations on Day 0 and Day 3.

Laboratory Test	Day 0	Day 3	Reference Range
White blood cell count	18.2 x 10^9^/L	4.1 x 10^9^/L	4.5-11 x 10^9^/L
Neutrophils	14.54 x 10^9^/L	3.47 x 10^9^/L	1.80-7.70 x 10^9^/L
Red blood cell count	4.74 x 10^12^/L	3.62 x 10^12^/L	4.3 - 5.7 x 10¹²/L
Hemoglobin	147 g/L	111 g/L	132 – 173 g/L
Platelets	300 x 10^9^/L	174 x 10^9^/L	140 – 400 x 10^9^/L
C-reactive protein	Not available	430 mg/L	< 3 mg/L

**Figure 1:**
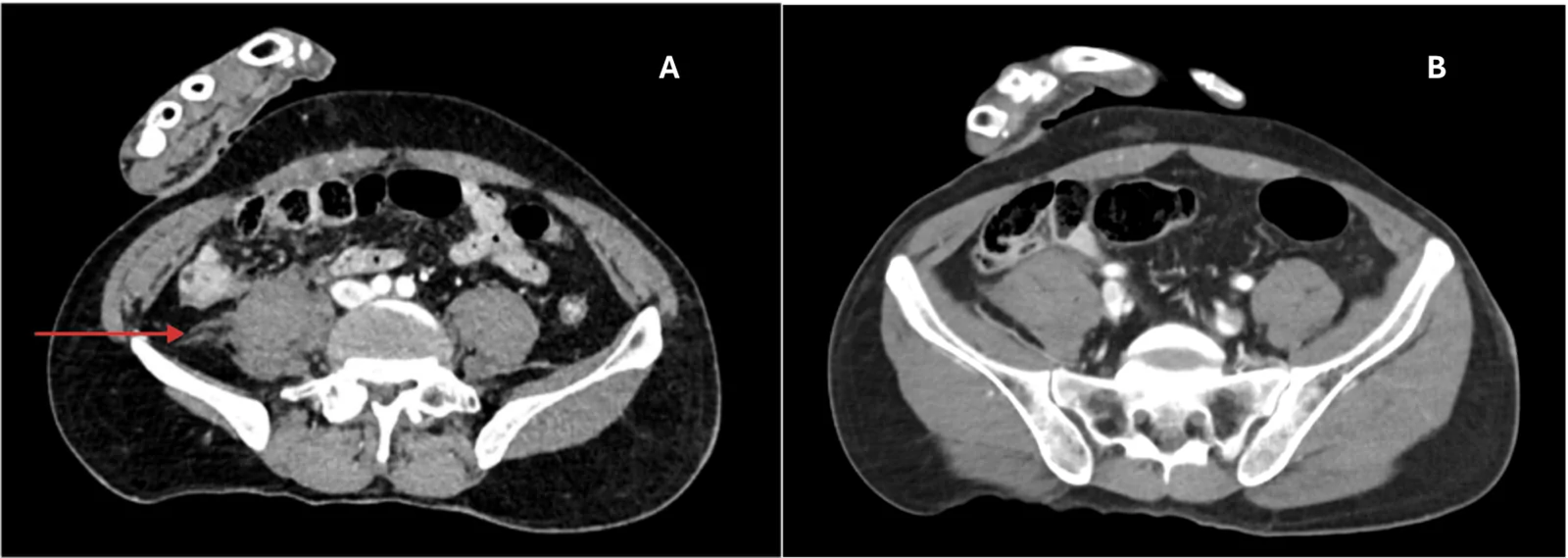
Initial CT findings on Day 0. (A) Right psoas intramuscular hematoma containing small air locules with surrounding soft tissue stranding (red arrow). (B) Representative axial CT image through the right lower abdominal/ileocecal region, with no obvious focal bowel wall discontinuity or free intraperitoneal air in this image. The initial CT examination demonstrated no pneumoperitoneum or definitive radiological evidence of bowel or mesenteric injury. The red arrow points at the right psoas intramuscular hematoma.

CT imaging demonstrated a right psoas intramuscular hematoma containing small air locules with surrounding soft tissue stranding (Figure 1). No pneumoperitoneum, mesenteric hematoma, or definitive radiological evidence of hollow viscus injury was identified. Associated injuries included a comminuted fracture of the surgical neck of the right humerus, a fracture of the right scapular spine, fractures of the left posterior fifth through tenth ribs, left hemopneumothorax with pulmonary contusions/lacerations, and displaced right L3-L4 transverse process fractures.

Given the patient’s hemodynamic stability, negative FAST examination, absence of definitive CT evidence of hollow viscus injury, and competing traumatic priorities, a decision was made for non-operative management with close observation. The patient received analgesia, intravenous antibiotics, tetanus prophylaxis, and underwent left chest tube insertion. The penetrating right lumbar wound was approximately 7 cm deep and involved the skin, subcutaneous tissue, and muscle. The wound was explored, underwent irrigation and surgical debridement, followed by layered closure and application of a pressure dressing. Serial abdominal examinations were continued during admission because of the initial abdominal symptoms.

**Figure 2:**
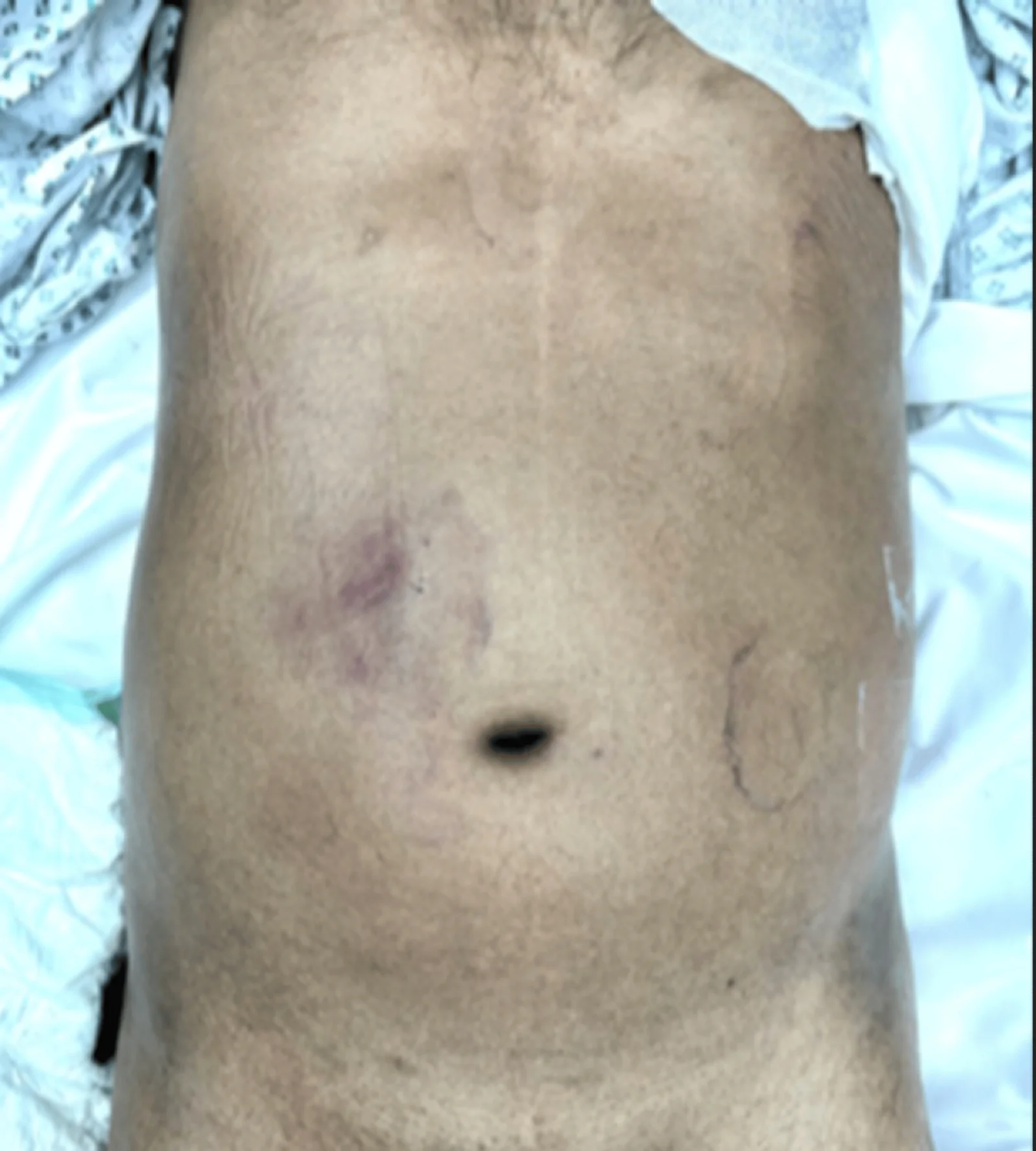
Newly developed abdominal wall bruising as seen pre-operatively on Day 3.

On Day 1, approximately 10 hours after presentation, he remained hemodynamically stable but continued to report right lower abdominal pain, with generalized guarding and right lower abdominal tenderness on examination. On Day 2, he remained clinically well and hemodynamically stable and was passing stool. Although he continued to report some abdominal pain earlier in the day, the abdomen was soft and non-tender. By the evening of Day 2, he denied abdominal pain and remained clinically well with a soft, non-tender abdomen. On Day 3, he developed worsening abdominal pain, abdominal distension, rigidity, new bruising over the right abdominal wall (Figure 2), and palpable subcutaneous crepitus. At the time of deterioration, his heart rate was 107 beats/min, blood pressure was 142/67 mmHg, respiratory rate was 16 breaths/min, temperature was 36.7°C, and oxygen saturation was 99% while receiving 1 L/min of oxygen via a nasal cannula.

**Figure 3:**
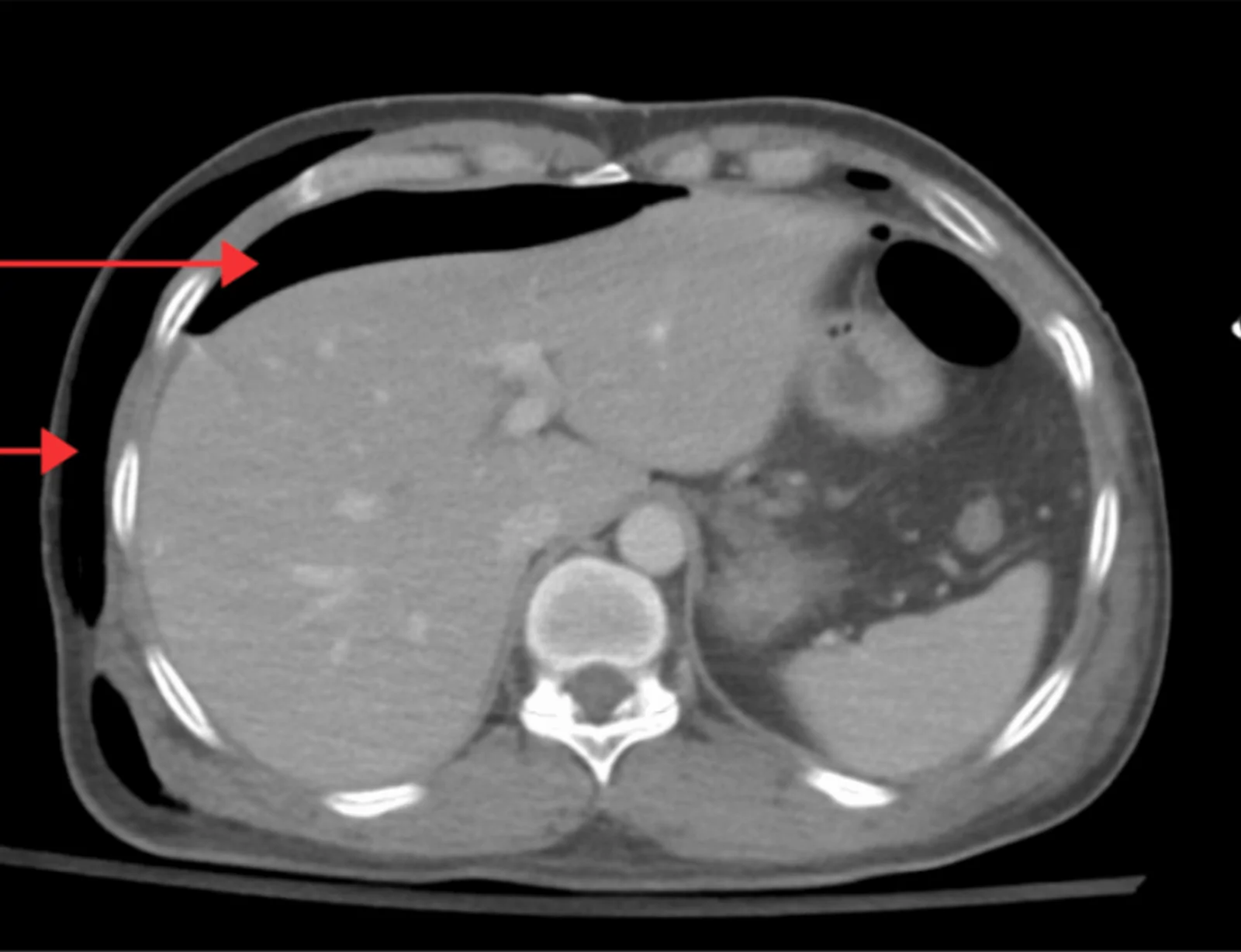
Repeat CT on Day 3 showing significant pneumoperitoneum anterior to the liver with subcutaneous emphysema of the right lateral abdominal wall. The top red arrow points at the significant pneumoperitoneum anterior to the liver The bottom red arrow points at the subcutaneous emphysema of the right lateral abdominal wall.

Urgent repeat CT demonstrated a large loculated right upper quadrant pneumoperitoneum anterior to the liver and hepatic flexure, with gas tracking along the anterior abdominal wall into the subcutaneous tissues (Figure 3). Associated right-sided ascites and peritoneal enhancement were consistent with peritonitis, with secondary thickening of the terminal ileum and extensive right abdominal wall emphysema. The radiological impression favored perforation at the hepatic flexure/proximal transverse colon. Repeat blood tests showed a fall in red blood cell count and hemoglobin, with markedly raised C-reactive protein (Table 1). Repeat lactate and blood gas analysis were not obtained on Day 3. Based on the clinical deterioration and CT findings, urgent surgical intervention was arranged.

**Figure 4:**
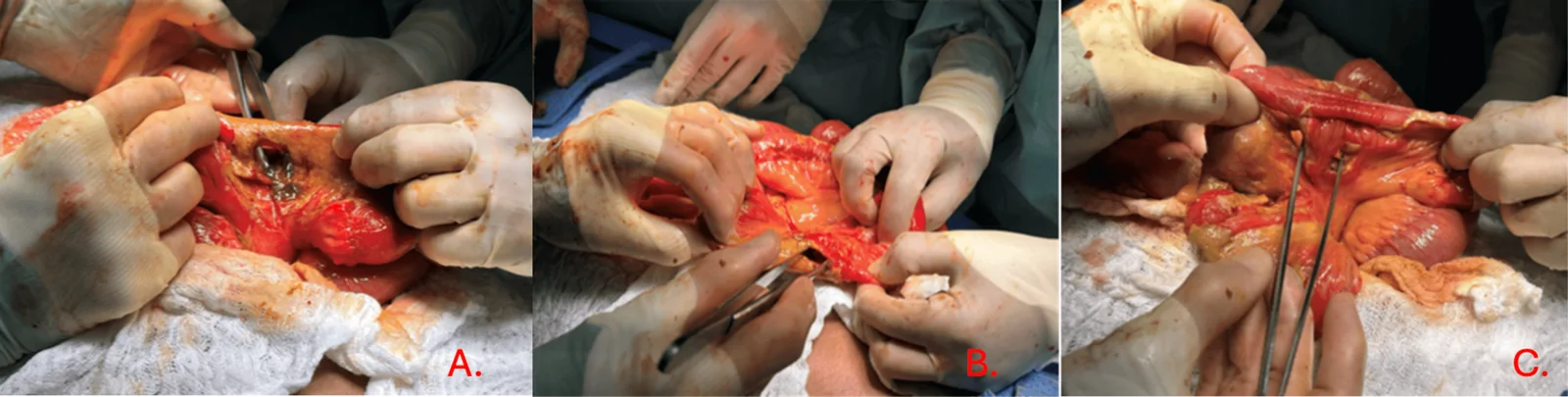
Intraoperative findings. (A) Two opposing full-thickness perforations involving the same small bowel segment. (B) Full-thickness small bowel perforation demonstrated with forceps. (C) Two adjacent mesenteric lacerations demonstrated with forceps.

Diagnostic laparoscopy was performed but was converted to laparotomy after identification of intestinal contents. Diffuse four-quadrant peritonitis was encountered. Multiple distal ileal perforations and mesenteric lacerations were identified (Figure 4).

No additional traumatic intra-abdominal organ injuries were identified. Segmental resection of the involved distal ileal loops was performed, followed by a mechanical side-to-side anastomosis using a GIA 55 stapler approximately 80 cm proximal to the ileocecal valve. The mesenteric defect was closed, extensive warm saline washout was performed, and a 19-F perianastomotic drain was placed. Primary anastomosis was selected due to viable bowel, hemodynamic stability, and controlled contamination [3,11,13,14]. The procedure lasted approximately 90 minutes. The operative record documented no significant blood loss, and no blood transfusion was required. The patient recovered well postoperatively without any significant complications. Oral intake resumed on Day 3, drains were removed on Day 5, and he was discharged on postoperative Day 9. Follow-up at two weeks showed transient vomiting and superficial wound contamination, managed conservatively as an outpatient. At the time of submission, follow-up duration was limited to two weeks since the patient moved back to their home country shortly after. Table 2 summarizes the course of events from admission until the latest follow-up.

**Table 2:** Timeline of the patient's course from admission until follow-up. FAST: Focused assessment with sonography for trauma

Day ​	Events ​	Details ​
Day 0 ​	Injury and ED presentation, initial assessment, initial CT, and initial interventions ​	Heavy object fell approximately 2-2.5 meters onto the right lumbar region, resulting in combined blunt and penetrating trauma. Hemodynamically stable, FAST negative. Psoas hematoma, no pneumoperitoneum, no definitive bowel injury. Chest tube insertion and wound debridement ​
Day 1	Observation ​	Hemodynamically stable with persistent right lower abdominal pain, generalized guarding, and right lower abdominal tenderness​
Day 2​	Clinical improvement​	Clinically well and hemodynamically stable, passing stool, with a soft and non-tender abdomen. Abdominal pain had resolved by the evening​
Day 3 ​	Clinical deterioration, repeat CT, surgical intervention​	Pain, distension, rigidity, crepitus on examination. CT shows pneumoperitoneum and peritonitis. Laparoscopy converted to laparotomy ​
POD 9 ​	Discharge ​	Stable ​
POD 15 ​	Follow-up ​	Minor complications managed conservatively as an outpatient. The patient moved back to their home country and was lost to follow-up

## Discussion

SBMI remain among the most challenging abdominal injuries to diagnose following trauma. Although CT is considered the imaging modality of choice in hemodynamically stable patients, its reported sensitivities vary considerably, and subtle injuries may be overlooked, particularly in the early stages following trauma [3,6]. Initial CT findings such as bowel wall thickening, mesenteric stranding, small amounts of free fluid, or limited extraluminal gas may be non-specific and can be difficult to distinguish from surrounding traumatic changes [6]. The present case was particularly challenging because of the combined blunt and penetrating mechanism, associated retroperitoneal injury, and multiple distracting thoracic and musculoskeletal injuries. While the initial CT demonstrated a right psoas hematoma with small intramuscular air locules and surrounding soft-tissue stranding, there was no definitive evidence of bowel wall disruption or mesenteric injury. The patient remained hemodynamically stable, and the FAST examination was negative. Severe generalized abdominal pain with diffuse tenderness and guarding was documented during the primary trauma survey; however, the subsequent examinations described a soft, non-distended abdomen with generalized or mild tenderness and no guarding. These fluctuating clinical findings, together with the absence of definitive bowel injury on the initial CT, contributed to the diagnostic difficulty.

The exact timing of bowel perforation in this patient cannot be established from the available clinical and radiological findings. Although no definitive hollow viscus injury was identified on the initial CT, an initially occult bowel injury may already have been present at the time of presentation. Alternatively, the injury may have evolved during the subsequent observation period. This distinction is important, as the clinical course demonstrates delayed recognition of bowel injury rather than proving that the perforations themselves developed several days after trauma.

Several mechanisms have been described for bowel injury that becomes clinically apparent after the initial trauma, including progressive mesenteric ischemia, expansion of intramural hematomas, evolving bowel wall necrosis, and initially occult full-thickness injury [3,11,14]. These mechanisms may explain delayed clinical presentation in some patients, although it is not possible to determine which, if any, occurred in the present case. The temporary improvement in this patient should therefore not be interpreted as evidence that bowel injury was absent during the initial assessment. The development of increasing abdominal pain, distension, abdominal wall bruising, subcutaneous emphysema, and radiological evidence of pneumoperitoneum ultimately prompted repeat assessment and operative intervention. Current trauma guidelines emphasize that no single CT finding can reliably exclude SBMI in high-risk patients. Instead, serial abdominal examinations, close observation, and repeat imaging when clinical status changes are recommended [3]. This approach was particularly important in the present case, where the repeat CT performed after clinical deterioration demonstrated clear evidence of peritonitis and pneumoperitoneum that had not been apparent initially. The repeat CT favored a perforation arising from the hepatic flexure or proximal transverse colon, but operative exploration revealed multiple distal ileal perforations and mesenteric lacerations. This discrepancy further illustrates the difficulty of accurately localizing traumatic hollow viscus injury on imaging alone. In retrospect, the severity of the initial abdominal pain and tenderness, together with the high-energy mechanism of injury and persistent concerning abdominal findings on reassessment approximately 10 hours after presentation, warranted continued suspicion for hollow viscus injury despite the non-definitive initial CT findings. Earlier repeat CT imaging or diagnostic laparoscopy could reasonably have been considered. However, by Day 2, the abdominal examination had normalized and the patient's pain had completely resolved, providing temporary clinical reassurance. Despite this improvement, an occult bowel injury could not be excluded, and repeat imaging within approximately 24 hours of the initial study may have identified evolving abnormalities before the development of generalized peritonitis. This represents an important learning point from the initial management of the case. Operative management was guided by intraoperative findings. Multiple distal ileal perforations and mesenteric lacerations were identified in the presence of diffuse four-quadrant peritonitis. Given the patient's hemodynamic stability, viable bowel, and the ability to achieve adequate source control, segmental resection with primary stapled anastomosis was performed [11,13,14]. This strategy is supported by contemporary trauma literature, which demonstrates favorable outcomes with primary anastomosis in appropriately selected patients [11,13,14]. This case highlights the importance of maintaining clinical suspicion for bowel injury following complex trauma even when initial imaging is non-definitive. Severe or persistent abdominal findings should not be dismissed simply because CT does not demonstrate a definite hollow viscus injury. It is also important to note that transient symptomatic improvement does not reliably exclude an occult injury. Continuous clinical assessment, timely repeat imaging, and consideration of diagnostic laparoscopy when concern persists may reduce delays in recognition and treatment.

## Conclusions

This report describes the delayed recognition of multiple small bowel perforations and mesenteric lacerations following combined blunt and penetrating lumbar trauma. Although the patient remained hemodynamically stable and initial imaging did not demonstrate definitive evidence of hollow viscus injury, he subsequently developed progressive abdominal symptoms and radiological signs of peritonitis that required urgent operative management. Segmental ileal resection with primary anastomosis resulted in a favourable short-term outcome.

Diagnosing SBMI can be particularly challenging when patients present with multiple associated injuries and initially non-specific findings. The clinical course demonstrates that bowel injury may remain clinically or radiologically occult during the initial assessment and that temporary symptomatic improvement does not reliably exclude significant injury. Careful serial examination, close observation, and a low threshold for repeat imaging remain essential in trauma patients with persistent or changing abdominal symptoms. When significant abdominal findings persist despite non-definitive initial CT imaging, early repeat imaging or diagnostic laparoscopy should be considered rather than relying on the initial imaging alone. Early recognition of deterioration and timely surgical intervention are crucial to minimise the morbidity associated with missed bowel injury.
